# Diabetes Mellitus

**Published:** 1846-04

**Authors:** 


10. *Diabetes Mellitus.*—Mr. Hodges, of Downpatrick, narrates the case of a girl, 17 years of age, laboring under diabetes mellitus, the result of a severe fall, in which he adopted, with apparent success, the nitrogenizing plan of treatment proposed by Dr. Barlow, in the Guy Hospital Reports, and also advised by M. Bouchardat. He prescribed the sesquicarbonate of ammonia in five grain doses every three hours, with coffee and bacon for breakfast, animal food and cruciferous vegetables for dinner, and further directed friction of the skin, and warm flannel clothing. The poor girl, who at the date of this prescription, was passing twenty-four pints of urine of the density 1.030, in the day and night, speedily improved; the secretion of urine diminished in four days to fourteen pints, the specific gravity continuing the same. This again fell in a few more days to eight pints, and that soon after to five, still, however, of the specific gravity of 1.030. By the end of the month, the qantity of urine was about four pints in the twenty-four hours—pulse 80; tongue clean, appetite natural, and the girl said she never enjoyed such good health. The report about six weeks afterwards was that she had gained strength and color, and considered herself quite recovered. The effect of the nitrogenizing treatment in this case was well marked, and such as to warrant its adoption in other cases. In this instance the density of the urine continued very nearly the same, until the sweet taste had disappeared, when it was reduced to 1.020, and the secretion exhibited the color and smell of healthy urine.—*Boston Med. and Sur. Journal.*

